# The inverse associations between composite-dietary-antioxidant-index and sarcopenia risk in US adults

**DOI:** 10.3389/fendo.2024.1442586

**Published:** 2024-09-17

**Authors:** Kang Wang, Qin Zhou, Zhongbiao Jiang, Shiping Liu, Hanfen Tang

**Affiliations:** ^1^ Department of Cardiology, Second Xiangya Hospital of Central South University, Changsha, China; ^2^ Department of General Surgery, Clinical Research Center for Breast Disease, Second Xiangya Hospital of Central South University, Changsha, China; ^3^ Department of Radiology, The Second Xiangya Hospital of Central South University, Changsha, China; ^4^ Department of Nutrition, The Second Xiangya Hospital of Central South University, Changsha, China

**Keywords:** observational study, composite dietary antioxidant index, sarcopenia, NHANES, public health

## Abstract

**Background:**

It remains unknown whether composite-dietary-antioxidant-index (CDAI) is associated with the risk of sarcopenia. This study investigated the association of CDAI with sarcopenia risk among general US adults.

**Methods:**

A total of 10,093 participants were enrolled in the National Health and Nutrition Examination Surveys (NHANES) from 6 survey cycles (2003-2004, 2005-2006, 2011-2012, 2013-2014, 2015-2016 and 2017-2018). Multivariate logistic regression was carried out to examine the relationship between CDAI and the risk of sarcopenia. Restricted cubic spline (RCS) curves were employed to analyze nonlinear relationships.

**Results:**

In a multi-variable logistic regression model adjusting for demographics, lifestyle, economic status and other dietary factors, higher CDAI score was related to a lower risk of sarcopenia among US adults. Compared the highest quartile of CDAI score with the lowest, the OR and 95%CI were 0.49 (0.31-0.75). Furthermore, the RCS demonstrated a linear dose-response relationship between CDAI and sarcopenia (*P*_non-linearity_=0.92). These results remained consistent across subgroups stratified by age, sex, physical activity, drinking status, body mass index (BMI), smoking habits, energy intake, and Healthy Eating Index (HEI) score. In addition, the favorable associations of CDAI were primarily attributed to Vitamin E intake.

**Conclusion:**

A higher CDAI score was associated with a lower risk of sarcopenia. According to these results, a greater adherence to CDAI may benefit sarcopenia prevention in adults.

## 1 Introduction

Sarcopenia, characterized by the accelerated loss of skeletal muscle function, strength, and mass, as individuals age, is a significant health concern globally (1). Currently, the prevalence rates of sarcopenia are estimated to vary from 10% to 27%, with severe sarcopenia affecting 2% to 9% of individuals (2), which is significantly associated with elevated risks of falls, functional impairments, frailty, and mortality (3–5).

Oxidative stress is a crucial factor in the development of sarcopenia (6). Impairment of the antioxidant defense mechanisms leads to excess reactive oxygen species (ROS) and oxidative stress within the organism. Excess ROS further destroys muscle cell structure, which may lead to muscle cell loss and decreased muscle strength (7, 8). Adopting a dietary pattern rich in antioxidant nutrients, such as Vitamin C, Vitamin E, carotenoids, selenium, flavonoids and some other plant phytochemicals may prevent the development of sarcopenia via influencing the oxidative damage. Composite dietary antioxidant index (CDAI) has been established as a credible and dependable nutritional instrument for evaluating antioxidants from 6 dietary sources: vitamins A, C, and E, selenium, carotenoids, and zinc (9). Prior studies have demonstrated beneficial associations between CDAI and multiple chronic diseases, such as hypertension (10), chronic kidney disease (CKD) (11), depression (12), cancer (13), coronary heart disease (14) and osteoporosis (15). However, no studies have so far examined the associations of CDAI scores with the risk of sarcopenia.

To explore this issue, we aimed to investigate the relationship between CDAI and sarcopenia risk. We hypothesized that a higher CDAI score was associated with a lower risk of sarcopenia.

## 2 Methods

### 2.1 Study population

The National-Health-and-Nutrition-Examination-Survey (NHANES) is a research initiative aimed at assessing the health and nutrition status of both children and adults across America. This survey is conducted annually and represents a nationally diverse sample of around 5,000 individuals from 15 counties. Its distinctiveness lies in its dual approach, combining structured interviews with comprehensive physical examinations. Interviews are conducted within respondents’ homes, while physical measurements are taken at specialized and well-equipped mobile centers, which travel to locations throughout the country (16).

**Figure 1:**
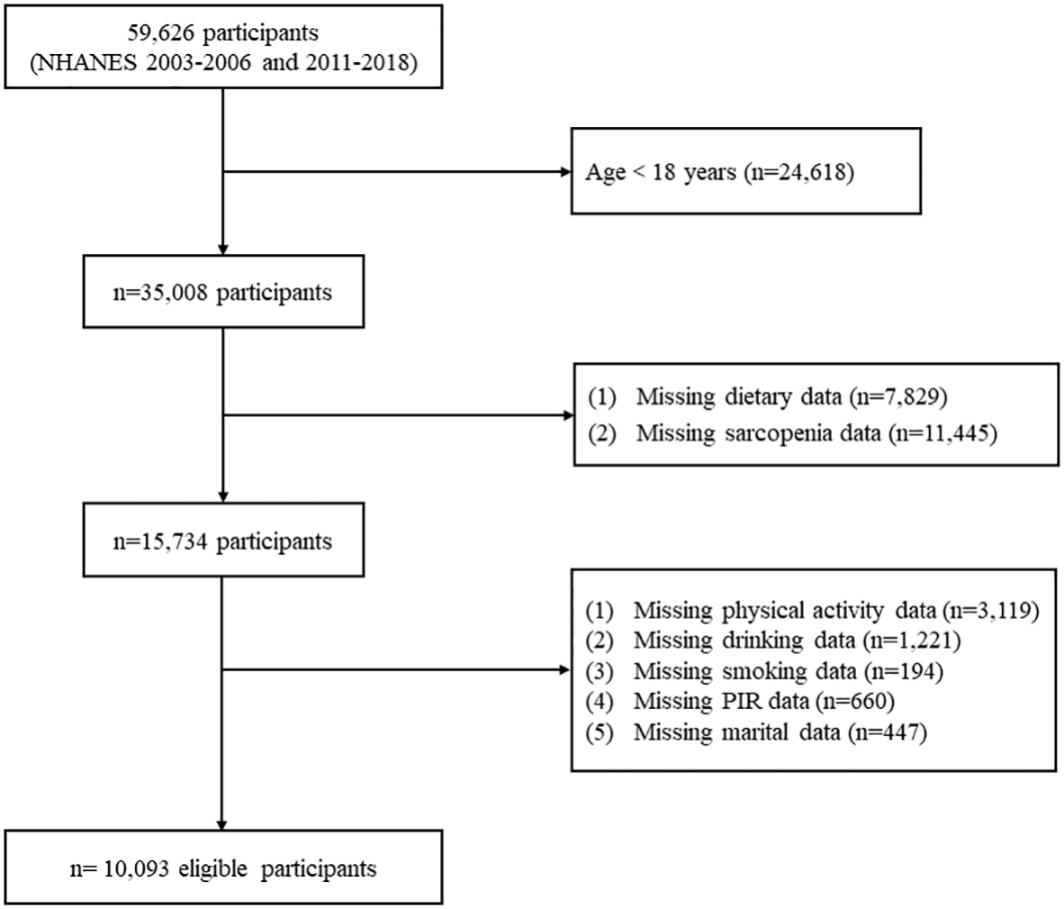
The flow chart of participant selection. PIR, poverty income ratio.

For the current study, we included 59,626 individuals from six NHANES survey cycles spanning from 2003 to 2018 (2003-2004, 2005-2006, 2011-2012, 2013-2014, 2015-2016, and 2017-2018). Exclusions were made for individuals under 18 years old (n=24,618), those lacking dietary data (n=7,829), and without sarcopenia data (n=11,445). Additionally, participants with missing covariate data (n=5,641), such as physical activity, smoking, drinking, poverty income ratio (PIR), and marital status, were further excluded. Finally, 10,093 participants were included in our study, representing approximately 104.7 million noninstitutionalized American citizens (**Figure 1**). The research protocol received approval from the Ethics Review Board at the National Center for Health Statistics, and all participants signed informed consent (17).

### 2.2 Assessment of dietary intake and CDAI

Food and nutrient intake data were acquired by professionals through two 24-hour dietary recall questionnaires at each survey cycle. The first questionnaire was administered face-to-face at the respondent’s home, followed by a second one conducted via telephone 3-10 days later (18). Dietary intake of antioxidants and total energy was calculated using the Food and Nutrient Database for Dietary Studies provided by the US Department of Agriculture (19). Antioxidants were sourced exclusively from dietary intake, excluding those from supplements or medications, and the average intake over two days was used for analysis. We included antioxidants data from 6 survey cycles (2003-2004, 2005-2006, 2011-2012, 2013-2014, 2015-2016 and 2017-2018).

To standardize antioxidant intake (including carotenoids, zinc, selenium, vitamins A, C, and E), we subtracted the gender-specific mean and divided by the gender-specific standard deviation. CDAI was calculated by summing the standardized intake of these antioxidants (9), which can be presented in the following formula:


$$\text{CDAI = }\sum_{i=1}^{6}\left(\text{Standard Intake of Antioxidant }i\right)$$

### 2.3 Ascertainment of sarcopenia

Sarcopenia was defined based on the guidelines established by FNIH and was characterized by appendicular lean mass (ALM) of <0.789 for males and <0.512 for females, after adjustment for body mass index (BMI) (20). ALM was determined as the sum of lean mass from the arm and leg evaluated through Dual-energy X-ray absorptiometry. Sarcopenia data were collected in the same cycle as the antioxidants data.

### 2.4 Assessment of covariates

Various covariates were collected through interview questionnaires, including age, gender, race, PIR, education, marital status, physical activity, smoking and drinking habits, daily energy intake, and healthy eating index (HEI). Marital status was classified into married, never married, and others. Educational levels were categorized as less than high school, high school, and more than high school. Drinking and smoking status are classified as current, former, or never. Physical activity was measured using total metabolic equivalent of task (MET) for one week. Based on the Global Physical Activity Questionnaire (GPAQ) (21), information was collected on different types of physical activity, such as work activity, transportation modes, and recreational activity. MET scores were assigned for each specific activity. Specifically, moderate and vigorous activities received 4 and 8 points, respectively. In addition, 4 points are assigned for transportation activity, including walking or bicycling (**Supplementary Table S1**). We calculated the MET (minutes/wk) for each specific activity by multiplying the duration of the specific activity by the corresponding score mentioned above, and then added up the MET (minutes/wk) for each specific activity to obtain the total MET (minutes/wk) (22). Daily energy intake was averaged over two days. The HEI was computed by summing scores for 13 vital dietary components, reflecting compliance to the 2015-2020 Dietary Guidelines for Americans. These comprise nine adequacy components (total fruits, whole fruits, total vegetables, green and beans, whole grains, dairy, total protein foods, seafood and plant proteins, and fatty acids) and four moderation components (refined grains, sodium, added sugars, and saturated fats). A higher HEI score reflects a higher diet quality (23).

Diabetes was diagnosed based on various criteria, including (i) random glucose content ≥11.1 mmol/L; (ii) HbA1c concentration ≥6.5%; (iii) fasting glucose level ≥7.0 mmol/L; (iv) oral glucose tolerance test ≥11.1 mmol/L; or (v) the use of antidiabetic drugs (24). The diagnostic criteria for hypertension included fulfilling one of the following conditions: (i) history of hypertension; (ii) taking antihypertensive medications; (iii) or with average systolic pressure ≥140 mmHg/average diastolic pressure ≥90 mmHg (25). Participants were diagnosed with CKD if the urine albumin/urine was ≥ 3 mg/mmol or if the glomerular filtration rate was < 60 ml/min/1.73 m^2^ for at least 3 months (26). Cancer was identified by asking “Have you ever been told by a doctor or other health professional that you had cancer or a malignancy of any kind?”.

### 2.5 Statistical analysis

The sample weights provided by NHANES were adjusted for different sampling rates, response rates, and different coverage rates among people in the sample. The sample weight for each respondent represents the estimated number of people in the target population, so that accurate national estimates can be obtained from the sample. All analyses incorporated sample weights “wtdr2d”. Baseline variable differences were assessed using Chi-Square and Student t tests. CDAI was divided into quartiles, and logistic regression model was employed to determine OR and 95%CI for the association of CDAI with sarcopenia. To address possible confounding, age (continuous, years) and sex (female and male) were adjusted in model 1. Model 2 further adjusted for race (White, Black, Hispanic, Mexican American and others), marital status (married, never married and others), education (more than high school, high school and less than high school), PIR (continuous), physical activity (continuous, MET-minutes/wk), smoking status (current, former and never), alcohol intake (current, former and never), BMI (continuous, kg/m^2^), and daily energy intake (continuous, kcal/d). Finally, we additionally adjusted for HEI (continuous) in model 3. Potential nonlinear relationships were explored using restricted cubic splines (RCS) regression with three nodes at the 10th, 50th, and 90th percentiles. In all spline analyses, exposure variables were treated with continuous data, and individuals with extreme first and last percentiles of 2.5 percent were excluded.

Furthermore, we conducted stratified analysis by several key risk factors, including age (<45, ≥45 years), gender (female, male), BMI (<30, ≥30 kg/m^2^), physical activity (<median, ≥median), alcohol intake (current, former, and never), smoking status (current, former, and never), daily energy intake (<median, ≥median), HEI score (<median, ≥median) and combined chronic diseases (no, yes) by adding an interaction term in model 3. The interaction was assessed in these stratified variables using the likelihood-ratio test.

Several sensitivity analyses were performed to test the robustness of the results. First, we further adjusted for chronic diseases, including diabetes, hypertension, CKD, and cancer. Second, we included populations with missing data on physical activity, smoking, and drinking, PIR, and marital status and utilized multiple imputations. Third, we additionally adjusted for specific dietary intake (including total fruit, total vegetable, whole grain, dairy, red meat, and fiber intake) instead of HEI score in model 3. Finally, we reanalyzed the data by excluding individuals with extreme energy intake (< 1000 kcal/d and > 5000 kcal/d).

All statistical tests were performed with R software (v4.3.1) and *P*<0.05 was deemed statistically significant.

## 3 Results

### 3.1 Baseline characteristics of study participants

**Table 1** displays the baseline characteristics of the participants. The mean age of these participants was 40.8 ± 0.3 years. Among them, 4,847 (48.5%) were females, and around 68.7% of the population identified as White. Compared with the participants without sarcopenia, those with sarcopenia tended to be older, have lower educational levels, income, and CDAI scores, and had higher BMI and a greater risk of chronic disease.

**Table 1:** Characteristics of the study population based on the presence of sarcopenia.

	Overall	No sarcopenia	With sarcopenia	*P*-value^a^
	n=10,093	n=9,215	n=878	
**CDAI**	0.15 (0.07)	0.25 (0.07)	-1.16 (0.15)	< 0.0001
**Age (years)**	40.8 (0.3)	40.3 (0.3)	47.0 (0.7)	< 0.0001
**Sex, %**				0.05
Female	4,847 (48.5)	4,455 (48.9)	392 (43.7)	
Male	5,246 (51.5)	4,760 (51.1)	486 (56.3)	
**Race, %**				< 0.0001
White	4,598 (68.7)	4,268 (69.7)	330 (56.3)	
Black	2,016 (9.9)	1,964 (10.4)	52 (3.7)	
Hispanic	693 (5.1)	605 (4.8)	88 (9.3)	
Mexican American	1,519 (8.6)	1,192 (7.5)	327 (22.8)	
Others	1,267 (7.7)	1,186 (7.7)	81 (7.8)	
**Education, %**				< 0.0001
Less than high school	573 (2.8)	412 (2.2)	161 (10.0)	
High school	3,269 (29.4)	2,906 (28.4)	363 (43.0)	
More than high school	6,251 (67.9)	5,897 (69.4)	354 (47.0)	
**Marital status, %**				0.056
Married	5,246 (54.2)	4,740 (53.9)	506 (58.6)	
Never married	2,353 (22.7)	2,220 (23.0)	133 (17.8)	
Others	2,494 (23.1)	2,255 (23.1)	239 (23.6)	
**Smoker, %**				0.082
Never	5,793 (56.6)	5,291 (56.6)	502 (56.7)	
Former	2,065 (21.8)	1,839 (21.6)	226 (25.6)	
Now	2,235 (21.5)	2,085 (21.8)	150 (17.7)	
**Drinker, %**				< 0.0001
Never	1,128 (9.2)	991 (8.6)	137 (16.6)	
Former	1,240 (9.9)	1,059 (9.4)	181 (16.0)	
Now	7,725 (80.9)	7,165 (81.9)	560 (67.4)	
**PIR**	3.1 (0.04)	3.2 (0.04)	2.6 (0.10)	< 0.0001
**BMI (kg/m^2^)**	28.0 (0.1)	27.6 (0.1)	34.0 (0.3)	< 0.0001
**Physical activity (MET-minutes/wk)**	3,629.2 (108.3)	3,656.2 (110.8)	3,261.2 (267.8)	0.144
**Daily energy intake (kcal/d)**	2,195 (12)	2,212 (13)	1,970 (37)	< 0.0001
**HEI score**	53.1 (0.3)	53.3 (0.3)	50.7 (0.5)	< 0.0001
**Diabetes, %**				< 0.0001
No	9,054 (92.6)	8,386 (93.4)	668 (80.8)	
Yes	1,039 (7.4)	829 (6.6)	210 (19.2)	
**Hypertension, %**				< 0.0001
No	7,019 (72.5)	6,558 (74.0)	461 (51.6)	
Yes	3,074 (27.5)	2,657 (26.0)	417 (48.4)	
C**KD, %**				< 0.0001
No	8,757 (89.0)	8,072 (92.3)	685 (85.3)	
Yes	981 (7.9)	823 (7.7)	158 (14.7)	
**Cancer, %**				0.248
No	9,560 (93.9)	8,744 (94.1)	816 (92.3)	
Yes	528 (6.0)	466 (5.9)	62 (7.7)	

Data expressed as mean [SD] or n (%). ^a^ *P*-value of the T-test or Chi-square test. CDAI, Composite Dietary Antioxidant Index; PIR, poverty income ratio; BMI, body mass index; MET, metabolic equivalent of task; HEI, healthy eating index; CKD, chronic kidney disease.

### 3.2 Relationship between CDAI and sarcopenia

Among the 10,093 participants, 8.7% (878/10,093) were diagnosed with sarcopenia. After adjustment for multiple covariates, such as demographics, lifestyle factors, economic status, energy intake, and HEI (Model 3), comparing with those in the lowest CDAI score, individuals with the highest CDAI score had a decreased risk of sarcopenia. The OR and 95%CI for extreme groups was 0.49 (0.31-0.75) (**Table 2**). Treating CDAI as a continuous variable, a one-point increase in CDAI score was related to a 5% lower risk of sarcopenia (**Table 2**). Further, we systematically excluded each of the six components from CDAI individually at a time, and observed that excluding vitamin E substantially attenuated the associations [0.64 (0.40-1.01)] (**Table 3**).

**Table 2:** Association of composite dietary antioxidant index and sarcopenia.

	Quartiles of CDAI				Continuous (per 1 score increase)
	Q1	Q2	Q3	Q4	
**Mean CDAI (SD)**	-4.12 (0.03)	-1.66 (0.02)	0.58 (0.02)	5.19 (0.10)	
**Case number (n)**	2,526	2,521	2,523	2,523	
**No. events (n)**	312	222	203	141	
**Model 1**	ref	0.61 (0.46,0.81)	0.62 (0.47,0.82)	0.28 (0.21,0.37)	0.89 (0.86,0.92)
**Model 2**	ref	0.76 (0.55,1.04)	0.81 (0.56,1.15)	0.43 (0.28,0.65)	0.94 (0.89,0.98)
**Model 3**	ref	0.79 (0.57,1.10)	0.88 (0.60,1.28)	0.49 (0.31,0.75)	0.95 (0.90,1.00)

Odds ratios (95% CIs) for risk of sarcopenia were analyzed using logistic regression models. Model 1: age (continuous, years) and sex (female and male). Model 2: Model 1 + race (White, Black, Hispanic, Mexican American and others), marital status (married, never married and others), education status (less than high school, high school and more than high school), PIR (continuous), physical activity (continuous, MET-minutes/wk), smoking status (never, former and now), alcohol intake (never, former and now), BMI (continuous, kg/m^2^), and daily energy intake (continuous, kcal/d). Model 3: Model 2 + healthy eating index (continuous). CDAI, Composite Dietary Antioxidant Index; PIR, poverty income ratio; BMI, body mass index; MET, metabolic equivalent of task; HEI, healthy eating index.

**Table 3:** Association with sarcopenia after exclusion of each one of 6 components from CDAI by one at a time.

	Quartiles of CDAI			
	Q1	Q2	Q3	Q4
CDAIa				
Model 1	ref	0.69 (0.51,0.94)	0.65 (0.48,0.87)	0.34 (0.24,0.49)
Model 2	ref	0.76 (0.53,1.07)	0.75 (0.50,1.12)	0.43 (0.27,0.68)
Model 3	ref	0.80 (0.56,1.13)	0.81 (0.53,1.22)	0.48 (0.30,0.77)
CDAIb				
Model 1	ref	0.69 (0.52,0.93)	0.56 (0.41,0.77)	0.27 (0.20,0.37)
Model 2	ref	0.79 (0.57,1.09)	0.67 (0.46,0.98)	0.38 (0.26,0.57)
Model 3	ref	0.81 (0.59,1.13)	0.71 (0.48,1.04)	0.41 (0.28,0.62)
CDAIc				
Model 1	ref	0.75 (0.56,1.01)	0.64 (0.46,0.89)	0.54 (0.37,0.78)
Model 2	ref	0.87 (0.62,1.22)	0.69 (0.46,1.04)	0.61 (0.38,0.96)
Model 3	ref	0.89 (0.63,1.25)	0.71 (0.47,1.08)	0.64 (0.40,1.01)
CDAId				
Model 1	ref	0.62 (0.47,0.83)	0.58 (0.44,0.77)	0.25 (0.18,0.34)
Model 2	ref	0.76 (0.56,1.04)	0.78 (0.55,1.09)	0.39 (0.25,0.60)
Model 3	ref	0.80 (0.58,1.10)	0.84 (0.59,1.20)	0.44 (0.28,0.69)
CDAIe				
Model 1	ref	0.71 (0.53,0.96)	0.68 (0.51,0.92)	0.37 (0.27,0.49)
Model 2	ref	0.87 (0.62,1.22)	0.86 (0.60,1.23)	0.53 (0.36,0.79)
Model 3	ref	0.93 (0.65,1.32)	0.95 (0.65,1.40)	0.62 (0.40,0.97)
CDAIf				
Model 1	ref	0.57 (0.42,0.76)	0.62 (0.45,0.85)	0.29 (0.20,0.40)
Model 2	ref	0.63 (0.45,0.88)	0.70 (0.47,1.04)	0.35 (0.23,0.54)
Model 3	ref	0.65 (0.46,0.92)	0.75 (0.50,1.13)	0.39 (0.24,0.62)

Odds ratios (95% CIs) for risk of sarcopenia were analyzed using logistic regression models. Model 1: age (continuous, years) and sex (female and male). Model 2: Model 1 + race (White, Black, Hispanic, Mexican American and others), marital status (married, never married and others), education status (less than high school, high school and more than high school), PIR (continuous), physical activity (continuous, MET-minutes/wk), smoking status (never, former and now), alcohol intake (never, former and now), BMI (continuous, kg/m^2^), and daily energy intake (continuous, kcal/d). Model 3: Model 2 + HEI (continuous).

CDAIa including vitamins C and E, zinc, selenium, and carotenoids.

CDAIb including vitamins A and E, zinc, selenium, and carotenoids.

CDAIc including vitamins A and C, zinc, selenium, and carotenoids.

CDAId including vitamins A, C and E, selenium, and carotenoids.

CDAIe including vitamins A, C and E, zinc, and carotenoids.

CDAIf including vitamins C and E, zinc, and selenium.

CDAI, Composite Dietary Antioxidant Index; PIR, poverty income ratio; BMI, body mass index; MET, metabolic equivalent of task; HEI, healthy eating index.

**Figure 2:**
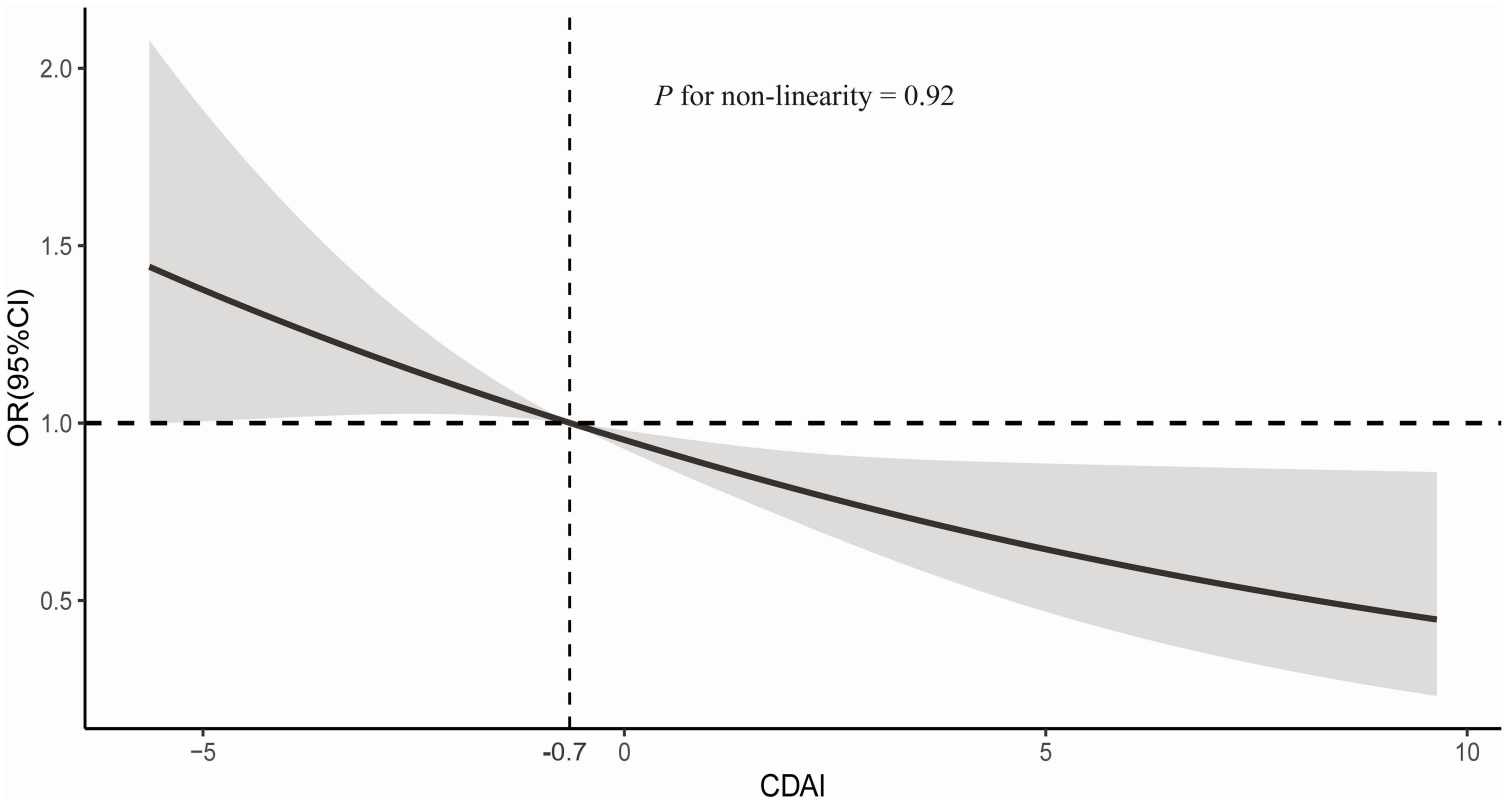
Dose-response relationships between CDAI with sarcopenia. Median CDAI score is reference standard. Odds ratio (OR) and 95%CI are based on logistic regression model adjusted for age (continuous, years) and sex (female and male), race (White, Black, Hispanic, Mexican American and others), marital status (married, never married and others), education status (less than high school, high school and more than high school), PIR (continuous), physical activity (continuous, MET-minutes/wk), smoking status (never, former and now), alcohol intake (never, former and now), BMI (continuous, kg/m^2^), and daily energy intake (continuous, kcal/d), healthy eating index (continuous). Solid lines indicate OR and shadow indicate 95%CI.

RCS analysis indicated a linear association between CDAI and sarcopenia (*P*_nonlinearity_=0.92). As depicted in **Figure 2**, the risk of sarcopenia decreases with higher CDAI scores. Specifically, the inflection point was identified at CDAI of approximately -0.7, which was associated with an OR of 1. Among the six antioxidant nutrients comprising CDAI, only vitamin A showed a nonlinear relationship with sarcopenia (**Supplementary Figure S1**).

### 3.3 Subgroup analysis

**Figure 3:**
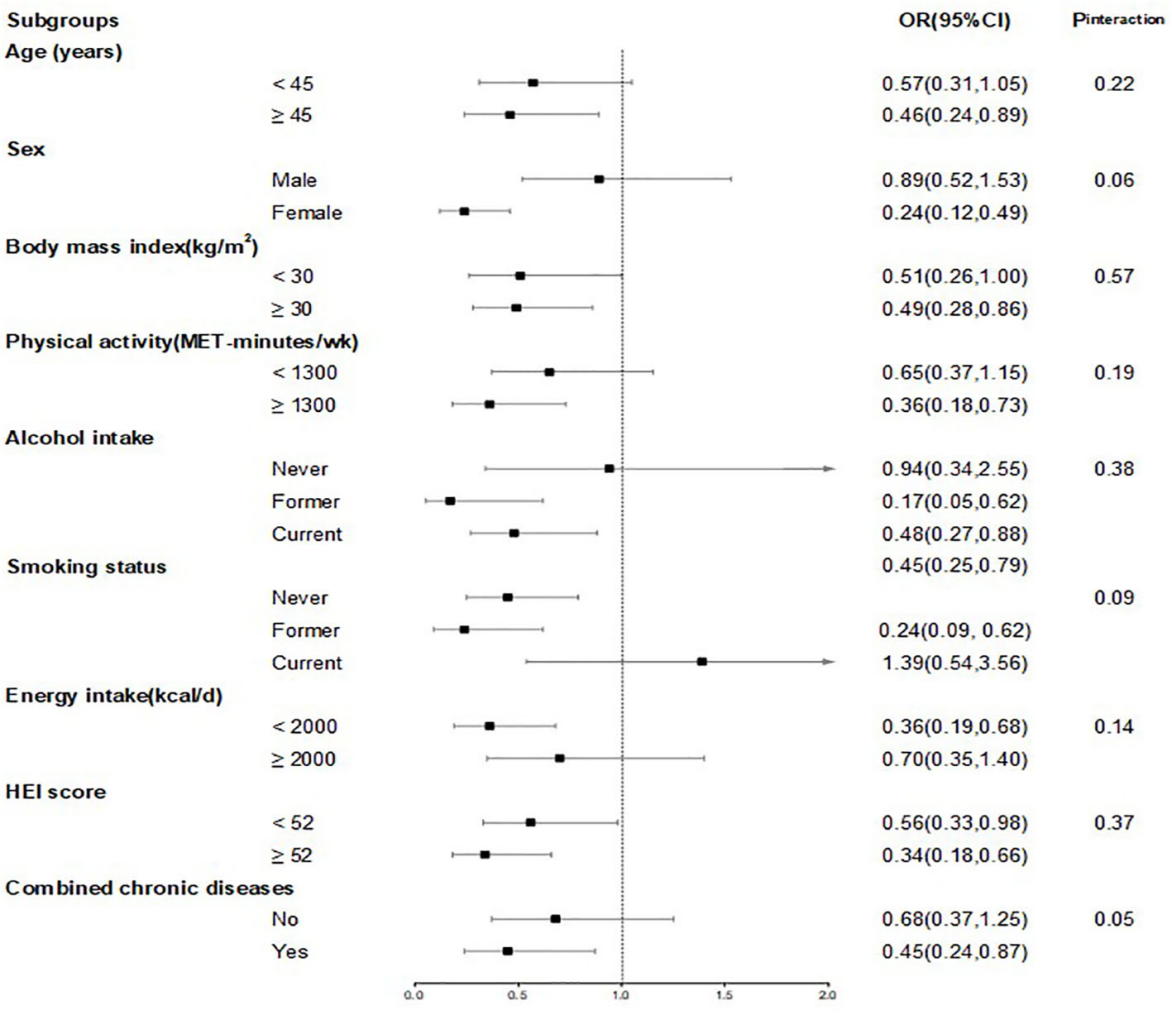
ORs and 95%CIs for CDAI and sarcopenia, stratified by several key risk factors. Model was adjusted for age (continuous, years) and sex (female and male), race (White, Black, Hispanic, Mexican American and others), marital status (married, never married and others), education status (less than high school, high school and more than high school), poverty income ratio (continuous), physical activity (continuous, MET-minutes/wk), smoking status (never, former and now), alcohol intake (never, former and now), BMI (continuous, kg/m^2^), and daily energy intake (continuous, kcal/d), healthy eating index (continuous). In each stratified analysis, the stratification variable was excluded in the adjustments. Chronic diseases include diabetes, hypertension, CVD and cancer. The OR and 95%CI of each subgroup in the figure from the group with the highest CDAI. Likelihood ratio tests were used for assessment of interaction, and two-sided P values (unadjusted for multiple comparisons) are reported.

In subgroup analysis, we observed associations across various strata, including sex (male, female), age (<45, ≥45 years), physical activity (<median, ≥median), alcohol intake (never, former and now), BMI (<30, ≥30 kg/m^2^), smoking status (never, former and now), HEI (<median, ≥median), energy intake (<median, ≥median) and combined chronic diseases (no, yes). None of the interaction P values between CDAI and these risk factors were statistically significant (all P>0.05) (**Figure 3**).

### 3.4 Sensitivity analyses

Firstly, after further adjusting for chronic diseases (including diabetes, hypertension, CKD and cancer), the results remained consistent (**Supplementary Table S2**). Secondly, when including populations with missing data on physical activity, smoking, drinking, PIR, and marital status and using multiple imputation, similar results were observed (**Supplementary Table S2**). Thirdly, after further adjusting for specific dietary intake (including fruit, vegetable, whole grain, dairy, red meat, and fiber) instead of HEI, the findings remained unchanged (**Supplementary Table S2**). Finally, to eliminate the potential impact of outliers, we reconstructed our model by excluding participants with extreme energy intake (<1000 and >5000 kcal/d). The results continued to show a decreasing risk of sarcopenia with increasing CDAI scores (**Supplementary Table S3**).

## 4 Discussion

In the present study, a higher CDAI score was associated with a lower risk of sarcopenia. The association was independent of age, sex, race, marital status, education, PIR, BMI, alcohol consumption, smoking behavior, physical activity, energy intake, and HEI. The association was also consistent across stratified groups by age, sex, BMI, physical activity, alcohol consumption patterns, smoking behavior, caloric intake, chronic diseases and HEI. Further, various sensitive analysis demonstrated similar results.

Advancements in the diagnosis and assessment of sarcopenia have been marked by the introduction of diverse methodologies and tools, enhancing our understanding and approach to muscle condition. The diagnosis of sarcopenia has been enriched by a variety of tools and methodologies that assess muscle mass, strength, and functionality (27). The Korean Genome and Epidemiology Study (KoGES) has proposed the muscle-to-fat ratio as a superior metric to BMI for evaluating body composition, particularly in overweight and obese individuals (28). The Framingham Heart Study has further advanced the field by highlighting the efficacy of computed tomography scans and establishing a systematic approach to interpret muscle metrics such as cross-sectional muscle area (CSMA), skeletal muscle index (SMI), skeletal muscle radio attenuation (SMRA), and skeletal muscle gauge (SMG) (29). Although, existing studies utilizing CDAI in the assessment sarcopenia are scarce, studies on related topics exist. A study that included 6,019 participants, also from the NHANES database, found a significant positive association between CDAI and handgrip strength (HGS). Interestingly, further gender-stratified analyses found this association to be significant in male but not female populations (30). Additionally, research involving adults with Metabolic Associated Fatty Liver Disease (MAFLD) using Dual-energy X-ray absorptiometry has revealed that higher CDAI scores are associated with a reduced risk of low muscle mass (31). The above studies support the finding of the current study that CDAI was positively linked to disease with decreased muscle strength, such as sarcopenia.

To the best of our knowledge, this current analysis is the initial effort to assess the associations of CDAI with sarcopenia risk. Previous studies have examined several healthful dietary eating indices, such as the Mediterranean diet (MED) index, the Healthy Eating Index 2015 (HEI-2015), the Alternative Healthy Eating Index 2010 (AHEI-2010), Japanese Food Guide Spinning Top (JFG-ST) and the oxidative balance score (OBS). A robust adherence to the Mediterranean Diet Score (MDS) has been linked to improved muscle outcomes, as evidenced by significant differences of 1.7% in fat-free mass percentage (FFM%) and a 9.6% rise in leg explosive power when comparing extreme quartiles of intake (32). According to the 2015-2020 Dietary Guidelines for Americans (DGA), individuals with the highest adherence to the HEI-2015 were 24% less likely to exhibit low grip strength than those in the lowest quartile among US adults (33). Nevertheless, an inverse correlation was observed between adherence to the AHEI-2010 and indigence of sarcopenia according to the 2019 European Working Group on Sarcopenia in Older People (EWGSOP2) criteria among women (34). The context of a 3-year Cohort Study focusing on elderly individuals living in community-dwelling, who were all above the age of 60, revealed that higher JFG-ST adherence scores were more likely to have greater SMI, specifically among the male participants (35). In other words, it is crucial to devise dietary guidelines specifically adopted to each country’s unique circumstances to prevent sarcopenia. More recently, after adjusting for potential confounders via the backward conditional method, no significant linkage was identified between the OBS and the likelihood of developing sarcopenia (36). These results corroborate the coherence of our study with the majority of previous studies, reinforcing the importance of considering dietary patterns in strategies aimed at promoting optimal muscle health throughout the aging process.

The underlying mechanisms of sarcopenia remain elusive despite being recognized as a multifactorial pathogenesis. This complex process involves oxidative stress, inflammation, mitochondrial dysfunction and reduced synthesis within the muscle tissue (37). While a myriad of risk factors, including advancing age, gender, physical activity, and dietary patterns are well-documented, the molecular mechanisms hinge on an aberrant imbalance between muscle protein synthesis and degradation (38). Crucial to muscle mass and function is the integrity of mitochondria. When compromised, they failed to generate reactive oxygen species (ROS) in a homeostatic manner, leading to a decline in cellular function and overall health (39). The consequential mitochondrial dysfunction is linked to impaired energy production and excessive ROS generation, which are key triggers for the phenotypic changes observed in sarcopenia patients. Moreover, dysregulated ROS production further correlates with elevated levels of inflammatory mediators, such as tumour necrosis factor-alpha (TNF-α), interleukin-6 (IL-6), nuclear factor-kappa B (NF-κB), and C-reactive protein (CRP), contributing to a pro-inflammatory state particularly in muscle tissue (40). Given the centrality of mitochondrial health in the pathogenesis of sarcopenia, interventions aimed at enhancing mitochondrial function, such as physical exercise and nutritional strategies, appear particularly promising in alleviating sarcopenia (41). In this context, our study has revealed a significant inverse correlation between higher CDAI scores and the likelihood of developing sarcopenia. This observation suggested that a diet abundant in antioxidants comprising vitamins C and E, as well as carotenoids may counteract the oxidative damage implicated in muscle degradation.

Individuals diagnosed with sarcopenia tend to have lower intake of essential nutrients such as selenium calcium, magnesium, and sodium compared to older adults with normal muscle function (42). Additionally, selenium deficiency has been linked to skeletal muscle disorders. A cross-sectional study found that higher selenium levels were associated with decreased physical limitations (43). Dietary factors are critical in modulating oxidative stress and protecting against ROS and reactive nitrogen species. Systematic reviews and meta-analyses revealed a correlation between consuming antioxidant-rich foods or antioxidant supplementation and a decreased risk of sarcopenia in individuals aged 55 and older (44). Similarly, data from the Korea-National-Health-and-Nutrition-Examination-Survey (2008–2011) demonstrated an opposite relationship between adequate intake of antioxidant nutrients and the incidence of sarcopenia in Korean adults (45). In a randomized, double-anonymized, placebo-controlled pilot study, supplementation with zinc, selenium, vitamin E, and vitamin C for 17 weeks did not significantly impact the two-minute walking test (2-MWT). However, the intervention did improve the maximum voluntary contractile force and sustained endurance limit time of the quadriceps muscles, possibly by improving the antioxidant defense system and decreasing oxidative stress (46). Overall, the literature suggests that individuals with sarcopenia often have nutrient deficiencies, highlighting the potential benefits of antioxidant nutrient interventions for the management of sarcopenia.

Despite limited research on CDAI and sarcopenia, previous research has explored the correlation between CDAI and various muscle-related conditions. Specifically, CDAI is positively correlated with HGS, with noted differences between sex (30). Additionally, higher CDAI scores have been related to a decreased risk of LAM in individuals with metabolic-associated fatty liver disease (31). Consistent with these findings, our study contributes to the growing body of evidence supporting an inverse correlation between CDAI and sarcopenia.

Vitamin C, a crucial water-soluble nonenzymatic antioxidant nutrient, has been found to positively correlate with skeletal muscle measurements among middle-aged and older individuals (47). Nevertheless, conflicting studies exist, with some indicating that vitamin C and E supplementation does not increase lower limb strength or reduce muscle damage in young athletes (48). According to NHANES data, dietary intake of vitamin E, selenium, and zinc is related to HGS in males, while only zinc intake is linked to HGS in females (30). The effectiveness of selenium supplementation in individuals with sarcopenia remains uncertain, as evidenced by observational studies (49). Prospective studies have demonstrated a favorable relationship between higher carotenoid intake and improved grip strength and walking speed among individuals in their middle and older years (50).

Conversely, a systematic review has highlighted the potential benefits of minerals such as magnesium and selenium for preventing and managing sarcopenia in the elderly (51). A positive correlation was observed between increased zinc intake and reduced risk of lower-extremity dysfunction and frailty in older adults, as indicated by a prospective study (52). Consequently, growing evidence suggests that sarcopenia can be prevented and managed through the consumption of vitamin E, vitamin C, and selenium. Subgroup analysis conducted in our study did not reveal any significant interactions among various risk factors, including age, gender, BMI, physical activity, alcohol consumption, smoking habits, chronic diseases and energy intake.

Interestingly, this study revealed that vitamin E significantly impacted the association between CDAI and sarcopenia. Furthermore, an investigation utilizing cross-sectional data originating from the fifth round of the ROAD study found that increased diet consumption of vitamin E and fats in the diet was associated with reduced sarcopenia (53). Another cross-sectional study indicated positive associations between intake of food-derived substances and plasma concentration of vitamin E in skeletal muscle mass, suggesting that dietary intake of vitamin E may play a significant role in preventing sarcopenia (54). Previous research has suggested that vitamin E deficiency may worsen sarcopenia, a condition often linked with aging, marked by decreased muscle strength and mass (55). A cross-sectional investigation employing data derived from the Korean-National-Health-and-Nutrition-Examination-Survey found that community-dwelling adults with lower serum vitamin E levels had weaker grip strength (56).

The notable strengths of our study include a substantial sample size and adjustment for various covariates. In addition, we carried out an array of sensitivity analyses which supported the robustness of our findings. However, our study had inherent limitations that need to be acknowledged. Firstly, our current analysis was conducted based on a cross-sectional study, which could not establish causal relationships. Secondly, the accuracy of 24-hour dietary questionnaires may be compromised by reliance on participants’ memory. Thirdly, our study was conducted among US populations, which may limit the broad applicability of our research findings across other racial/ethnic or socioeconomic groups. Finally, as with all observational studies, we cannot guarantee the absence of any residual confounding elements despite adjusting for dietary, lifestyle and medical history factors in our analysis.

## 5 Conclusion

Our data indicated a beneficial association between CDAI and the incidence of sarcopenia among US adults. Whether the beneficial association exists in other populations warrants further investigation.

## Data Availability

Publicly available datasets were analyzed in this study. This data can be found here: https://www.cdc.gov/nchs/nhanes/index.htm.
