# Supplementary material for: The inverse associations between composite-dietary-antioxidant-index and sarcopenia risk in US adults
Source: Front Endocrinol (Lausanne). 2024 Sep 17;15:1442586. doi: 10.3389/fendo.2024.1442586 (PMC11442324; doi:10.3389/fendo.2024.1442586)
Supplement: Supplementary file 1 [file Table1.docx]

**Online Supplementary Materials**

**The inverse associations between composite-dietary-antioxidant-index and sarcopenia risk in US adults**

Kang Wang, Qin Zhou, Zhongbiao Jiang, Shiping Liu, Hanfen Tang

**Supplementary Figure 1**: Dose-response relationships of six components of CDAI with sarcopenia 2

**Supplementary Table 1**: MET scores of physical activities 3

**Supplementary Table 2**: ORs and 95%CIs for sarcopenia by quartiles of CDAI using alternative models of adjustment 4

**Supplementary Table 3**: ORs and 95%CIs for sarcopenia by quartiles of CDAI after excluding participants with extreme energy intake 4


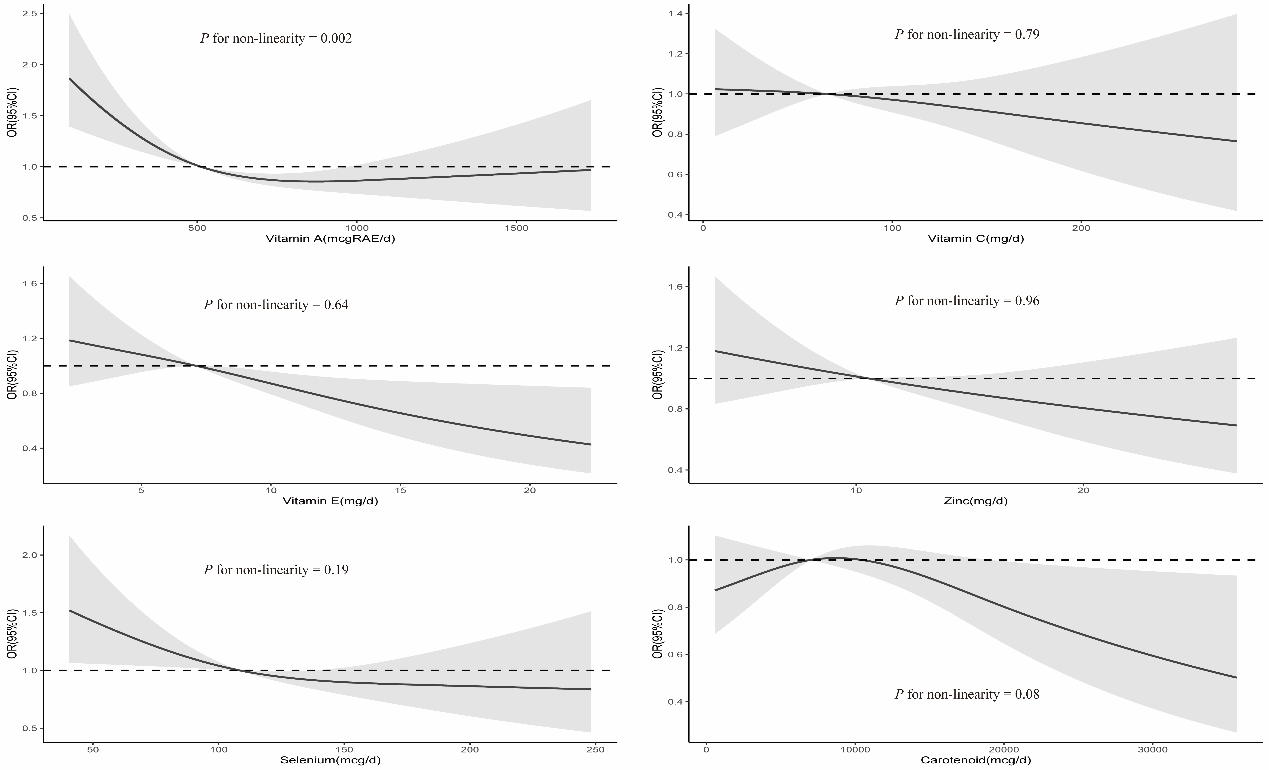


**Supplementary Figure 1. Dose-response relationships of six components of CDAI with sarcopenia.** Median CDAI score is reference standard. Odds ratio (OR) and 95%CI are based on logistic regression model adjusted for age (continuous, years) and sex (female and male), race (White, Black, Hispanic, Mexican American and others), marital status (married, never married and others), education status (less than high school, high school and more than high school), PIR (continuous), physical activity (continuous, MET-minutes/wk), smoking status (never, former and now), alcohol intake (never, former and now), BMI (continuous, kg/m^2^), and daily energy intake (continuous, kcal/d), HEI (continuous). Solid lines indicate OR and shadow indicate 95%CI. Abbreviations: PIR, poverty income ratio; BMI, body mass index; MET, metabolic equivalent of task; HEI, healthy eating index.

**Supplementary Table 1. MET scores of physical activities.**

|  | **MET score** | | |
| --- | --- | --- | --- |
|  | 0 | 4 | 8 |
| Work activity^+^ | No | Moderate | Vigorous |
| Transportation modes | others | Walking or bicycling |  |
| Recreational activities^++^ | No | Moderate | Vigorous |

Abbreviations: MET, metabolic equivalent of task;

^+^ including carrying or lifting loads, digging or construction work and so on;

^++^ including bicycling, swimming, volleyball, running, basketball and so on.

**Supplementary Table 2.** **ORs and 95%CIs for sarcopenia by quartiles of CDAI using alternative models of adjustment.**

|  | **Quartiles of CDAI** | | | |
| --- | --- | --- | --- | --- |
|  | **Q1** | **Q2** | **Q3** | **Q4** |
| **Model 3** | 1 (reference) | 0.79(0.57,1.10) | 0.88(0.60,1.28) | 0.49(0.31,0.75) |
| **Model 3b** | 1 (reference) | 0.79(0.59,1.06) | 0.94(0.65,1.34) | 0.51(0.33,0.80) |
| **Model 3c** | 1 (reference) | 0.91(0.71,1.17) | 0.92(0.68,1.26) | 0.54(0.38,0.76) |
| **Model 3d** | 1 (reference) | 0.81(0.58,1.12) | 0.93(0.63,1.36) | 0.53(0.33,0.85) |

Model 3(original model) adjusted for age (continuous, years) and sex (female and male), race (White, Black, Hispanic, Mexican American and others), marital status (married, never married and others), education status (less than high school, high school and more than high school), PIR (continuous), physical activity (continuous, MET-minutes/wk), smoking status (never, former and now), alcohol intake (never, former and now), BMI (continuous, kg/m^2^), and daily energy intake (continuous, kcal/d), HEI (continuous).

Model 3b: Model 3 + diabetes, hypertension, CKD, and cancer (yes or no).

Model 3c included populations with missing data on physical activity, smoking, and drinking, PIR, and marital status and used multiple imputation.

Model 3d: Model 2 + specific dietary intake (including fruit (continuous, cup/d), vegetable (continuous, cup/d), whole grain (continuous, oz/d), dairy (continuous, cup/d), red meat (continuous, oz/d) and fiber (continuous, g/d)).

Abbreviations: PIR, poverty income ratio; BMI, body mass index; MET, metabolic equivalent of task; HEI, healthy eating index; CKD: chronic kidney disease.

**Supplementary Table 3.** **ORs and 95%CIs for sarcopenia by quartiles of CDAI after excluding participants with extreme energy intake (< 1000 and > 5000 kcal/d).**

|  | **Quartiles of CDAI** | | | |
| --- | --- | --- | --- | --- |
|  | **Q1** | **Q2** | **Q3** | **Q4** |
| **Model 1** | 1 (reference) | 0.58(0.43,0.78) | 0.61(0.46,0.82) | 0.28(0.21,0.37) |
| **Model 2** | 1 (reference) | 0.69(0.49,0.98) | 0.76(0.52,1.10) | 0.40(0.26,0.62) |
| **Model 3** | 1 (reference) | 0.72(0.51,1.03) | 0.83(0.56,1.22) | 0.46(0.29,0.72) |

Model 1: age (continuous, years) and sex (female and male).

Model 2: Model 1 + race (White, Black, Hispanic, Mexican American and others), marital status (married, never married and others), education status (less than high school, high school and more than high school), PIR(continuous), physical activity (continuous, MET-minutes/wk), smoking status (never, former and now), alcohol intake (never, former and now), BMI (continuous, kg/m^2^), and daily energy intake (continuous, kcal/d).

Model 3: Model 2 + HEI (continuous).

Abbreviations: PIR, poverty income ratio; BMI, body mass index; MET, metabolic equivalent of task; HEI, healthy eating index.
